# Change in maternal income status following stillbirth, neonatal death and severe neonatal morbidity

**DOI:** 10.1186/s40748-026-00249-8

**Published:** 2026-01-21

**Authors:** Jennifer A. Jairam, Hilary K. Brown, Christina Diong, Howard Berger, Jun Guan, Eyal Cohen, Joel G. Ray

**Affiliations:** 1 Department of Medicine Research, St. Michael’s Hospital, Toronto, ON Canada; 2 ICES, Toronto, ON Canada; 3 Department of Health and Society, University of Toronto, Toronto, ON Canada; 4 Department of Medicine, St. Michael’s Hospital, 30 Bond St, Toronto, ON M5B 1W8 Canada; 5 Department of Obstetrics and Gynaecology, St. Michael’s Hospital, Toronto, ON Canada; 6 Child Health Evaluative Sciences, SickKids Research Institute, Toronto, ON Canada; 7 Edwin S. H. Leong Centre for Healthy Children, University of Toronto, Toronto, ON Canada; 8 Keenan Research Centre, St. Michael’s Hospital, Toronto, ON Canada

**Keywords:** Stillbirth, Neonatal morbidity, Neonatal death, Neighbourhood income

## Abstract

**Aim:**

We evaluated whether stillbirth, neonatal death or severe neonatal morbidity is associated with a mother’s change in residential neighbourhood income between two consecutive births.

**Methods:**

This population-based cohort included all mothers in Ontario, Canada with two consecutive births at 20–42 weeks’ gestation, 2003–2023. The study exposure at the first birth was: (i) livebirth unaffected by severe neonatal morbidity or neonatal death (referent); (ii) livebirth with severe neonatal morbidity but no neonatal death; (iii) livebirth with neonatal death; and (iv) stillbirth. The study outcome was a mother’s change in residential neighbourhood income quintile (Q) between two consecutive births: (i) downward income mobility, (ii) upward income mobility, or (iii) persistently residing in the lowest income Q1 at each birth. -- each relative to iv) no change in neighbourhood income Q between births (referent).

**Results:**

There were 720,119 mothers included. Among those initially residing in income Q2-5, relative to those with an unaffected livebirth, there was a higher likelihood of downward income mobility between births if their child was affected by non-fatal severe neonatal morbidity (aOR 1.05, 95% CI 1.02-1.08). Among mothers initially residing in income Q1, the aOR for remaining in income Q1 was 1.05 (95% CI 1.01-1.08) following a livebirth affected by severe neonatal morbidity, 1.29 (95% CI 1.12-1.48) after a neonatal death, and 1.35 (95% CI 1.24-1.46) after a stillbirth – each compared to mothers with an unaffected birth.

**Conclusions:**

Mothers with a newborn affected by severe morbidity were more likely to have a decline in neighbourhood income Q, or to persist in the lowest-income area. Those experiencing stillbirth or neonatal death were more likely to remain in a lowest-income neighbourhood or have no income mobility.

**Supplementary Information:**

The online version contains supplementary material available at 10.1186/s40748-026-00249-8.

## Introduction

**Fig. 1:**
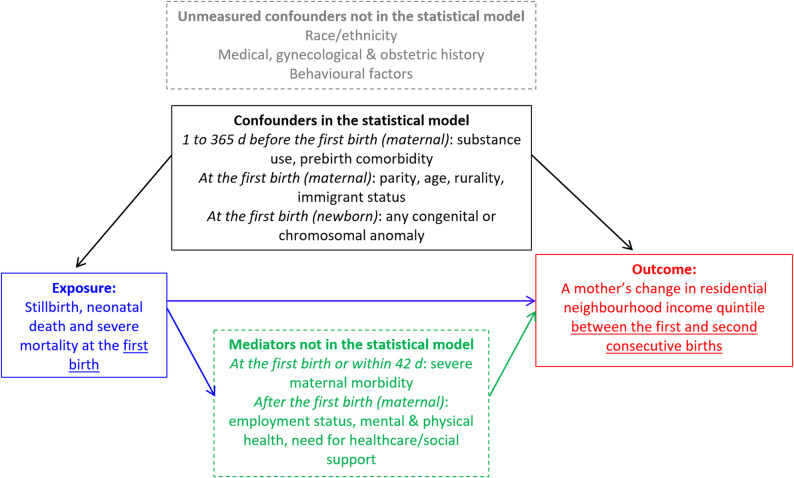
Conceptual diagram used in the current study for how stillbirth, neonatal death or severe morbidity occurring at the first birth might be associated with a mother’s subsequent change in residential neighbourhood income quintile between the first and consecutive second births

A birth can create or exacerbate income loss for parents due to child healthcare expenses, [1] workforce absenteeism [2] and the cost of childrearing [3] – together economically termed the ‘child penalty’ [4] – and which can further influence residential affordability. This could be most taxing on women residing in low-income neighbourhoods, with financial instability prior to childbirth [5]. Research indicates that a pregnancy culminating in a stillbirth, neonatal death, or severe neonatal morbidity is associated with maternal postpartum complications, [6] along with profound short- and long-term psychological, social, and economic impacts for both parents [7–9]. Adverse pregnancy events are also more prevalent among those affected by social inequities [7, 10]. Hence, it is plausible that economic challenges may further differ if a woman experiences fetal or neonatal loss, marked by bereavement but no ongoing child-rearing costs thereafter, or has a newborn with transient morbidity followed by a potentially higher economic burden [11] (Fig. 1).

This study assessed whether a stillbirth, neonatal death or severe neonatal morbidity is associated with a mother’s subsequent change in residential neighbourhood income between two consecutive births.

## Methods and data analysis

This population-based cohort study was completed using linked administrative datasets in Ontario, Canada (Table S1), where universal healthcare exists. Included were all mothers with two consecutive births at 20–42 weeks’ gestation, from April 1, 2003, to March 31, 2023 (Table S2). The online Supporting Information contains details of all methods, study variables, and analyses. The STROBE reporting guideline was followed.

The study exposure ─ an adverse perinatal event -- comprised four mutually exclusive groups at the **first** of two consecutive births in the study period: (i) livebirth unaffected by severe neonatal morbidity or neonatal death (referent); (ii) livebirth with severe neonatal morbidity but no neonatal death; (iii) livebirth with neonatal death; and (iv) stillbirth. Severe neonatal morbidity and neonatal death were assessed from the index birth hospitalization up to 27 days thereafter, with severe morbidity defined using a validated composite measure of serious newborn health issues [12]. 

The study outcome was a mother’s change in residential neighbourhood income quintile (Q) between two consecutive births, representing four exclusive mobility patterns: (i) downward income mobility, (ii) upward income mobility, or (iii) persistently residing in the lowest income Q1 at each birth -- each relative to (iv) no change in neighbourhood income Q between births (referent). The latter two exposure groups were categorized separately as they likely comprise mothers with different economic circumstances, especially since persistent residence in a lowest income neighbourhood would be indicative of long-term area-level economic instability.

Descriptive statistics (i.e., mean and median values and proportions) were contrasted by the adverse perinatal exposure states at the first birth. Multinomial logistic regression generated odds ratios (aORs) and 95% confidence intervals (CI) for the primary outcome of a mother’s change in neighbourhood income Q between two consecutive births in relation to the study exposure of an adverse perinatal event at the first birth.

Statistical models were adjusted for the following maternal and newborn characteristics: substance use (opioid, cocaine, stimulant, or alcohol use) 1 year before the first birth, number of prebirth comorbidities 1 year before the first birth, maternal age at the first birth, livebirth parity at the first birth, rurality at the first birth, immigrant status, and any newborn congenital or chromosomal anomaly identified within the first birth hospitalization.

## Results

Of the 720,119 mothers, whose mean (SD) age was 28.6 (4.8) years at the first recorded birth, 669,232 (92.9%) had a livebirth without severe neonatal morbidity or neonatal death, 44,251 (6.1%) had a livebirth with severe neonatal morbidity but no neonatal death, 1756 (0.24%) had a livebirth with neonatal death, and 4880 (0.68%) had a stillbirth (Table 1).

**Table 1:** Maternal and fetal/newborn characteristics by pregnancy outcomes at the first birth among 720,119 women who had two consecutive births in Ontario, Canada, 2003 to 2023. Data are presented as a number (%) unless otherwise indicated

Characteristic	Livebirth without severe neonatal morbidity or neonatal death (*N* = 669,232)	Livebirth with severe neonatal morbidity without neonatal death^a^ (*N* = 44,251)	Livebirth with neonatal death^b^ (*N* = 1756)	Stillbirth^c^ (*N* = 4880)
**Maternal, 1 to 365 days before the first birth**				
Number of pre-birth comorbidities^d^				
* 0–2*	619,320 (92.5)	39,848 (90.1)	1528 (87.0)	4349 (89.1)
* ≥ 3*	49,912 (7.5)	4403 (10.0)	228 (13.0)	531 (10.9)
Substance use (opioid, cocaine, stimulant or alcohol use)^e^	18,352 (2.7)	2028 (4.6)	87 (5.0)	239 (4.9)
**Maternal**,** at the first birth**				
Mean (SD), Age, years	28.6 (4.8)	28.7 (4.9)	29.4 (5.4)	30.0 (5.4)
Neighbourhood income quintile, Q				
* Q1 (Lowest)*	141,061 (21.1)	9252 (20.9)	450 (25.6)	1276 (26.2)
* Q2*	135,799 (20.3)	9012 (20.4)	415 (23.6)	1000 (20.5)
* Q3*	140,757 (21.0)	9510 (21.5)	348 (19.8)	1019 (20.9)
* Q4*	141,685 (21.2)	9461 (21.4)	321 (18.3)	925 (19.0)
*Q5 (Highest)*	109,930 (16.4)	7016 (15.9)	222 (12.6)	660 (13.5)
Parity				
* 0–1*	624,654 (93.3)	41,969 (94.8)	1552 (88.4)	4260 (87.3)
* ≥ 2*	44,578 (6.7)	2282 (5.2)	204 (11.6)	620 (12.7)
Rural residence	68,070 (10.2)	4415 (10.0)	180 (10.3)	476 (9.8)
Non-refugee immigrant	159,328 (23.8)	9558 (21.6)	454 (25.9)	1373 (28.1)
**Fetus or newborn**,** at the first birth**				
Female sex	328,950 (49.1)	18,662 (42.2)	785 (44.7)	61 (1.2)
* Sex missing*	0 (0.0)	0 (0.0)	0 (0.0)	4745 (97.2)
Gestational age at birth, mean (SD), weeks	39.2 (1.4)	37.3 (3.7)	29.2 (7.4)	30.2 (7.1)
Preterm livebirth or stillbirth < 37 weeks’ gestation	27,818 (4.2)	13,057 (29.5)	1254 (71.1)	3441 (70.5)
Any congenital or chromosomal anomaly	17,801 (2.7)	6306 (14.3)	544 (31.0)	Not reported
**Median IQR**,** number of months between the first & second births**,** years**	32 (24–47)	32 (24–46)	20 (14–32)	19 (14–30)

^a^Arising in the index birth admission or within 27 days thereafter

^b^Arising in the index birth admission or within 27 days thereafter

^c^A fetal death arising *in utero*, or a newborn with no signs of life at birth, at ≥ 20 weeks’ gestation

^d^Total number of Johns Hopkins Adjusted Clinical Group (ACG)^®^ System Aggregated Diagnosis Groups (ADG) (excluding “pregnancy” diagnosis), 1 to 365 days before the first birth hospitalization

^e^Type of substance use, 1 to 365 days before the first birth hospitalization is suppressed due to small cell sizes ≤ 5

Among mothers initially residing in income areas Q2-Q5, relative to those with an unaffected livebirth, there was a higher likelihood of downward income mobility between births if their child was affected by non-fatal severe neonatal morbidity (aOR 1.05, 95% CI 1.02–1.08), with no tendencies for upward income mobility (Table 2). However, significantly lower aORs were observed for experiencing downward or upward income mobility between births if they had a neonatal death or a stillbirth.

**Table 2:** Odds of a mother exclusively experiencing downward or upward income mobility between two consecutive births, or otherwise persistently residing in neighbourhood income quintile 1 (Q1) between two successive births, each in relation to having a newborn affected by a stillbirth, neonatal death or severe neonatal morbidity at the first recorded birth. Data are limited to women who had two recorded consecutive Singleton hospital births at 20^0/7^ to 42^0/7^ weeks’ gestation in Ontario, Canada, 2003 to 2023

	Outcome occurring between consecutive births					
	Downward income mobility		Upward income mobility		Persistently residing in Q1	
**Exposure at the first birth**	**No. (%) with** **outcome**	**Adjusted** **odds ratio** **(95% CI)** ^e, f^	**No. (%) with** **outcome**	**Adjusted** **odds ratio** **(95% CI)** ^e, f^	**No. (%) with** **outcome**	**Adjusted** **odds ratio** **(95% CI)** ^e, f^
*Livebirth without severe neonatal morbidity or neonatal death* *(N = 669,232)* ^a^	122,443 (18.3)	1 (Reference)	168,199 (25.1)	1 (Reference)	79,465 (11.9)	1 (Reference)
*Livebirth with severe neonatal morbidity without death* *(N = 44*,*251)*^b^	8343 (18.9)	**1.05 (1.02 to 1.08)**	11,000 (24.9)	1.01 (0.99 to 1.04)	5241 (11.8)	**1.05 (1.01 to 1.08)**
*Livebirth with neonatal death* *(N = 1756)* ^c^	294 (16.7)	**0.86 (0.75 to 0.99)**	326 (18.6)	**0.71 (0.62 to 0.81)**	301 (17.1)	**1.29 (1.12 to 1.48)**
*Stillbirth * *(N = 4880)* ^d^	734 (15.0)	**0.77 (0.71 to 0.84)**	837 (17.2)	**0.64 (0.59 to 0.69)**	893 (18.3)	**1.35 (1.24 to 1.46)**

^a^299,125 (44.7%) of mothers had no income mobility between two consecutive births, except those shown with persistent Q1

^b^19,667 (44.4%) of mothers had no income mobility between two consecutive births, except those shown with persistent Q1

^c^835 (47.6%) of mothers had no income mobility between two consecutive births, except those shown with persistent Q1

^d^2416 (49.5%) of mothers had no income mobility between two consecutive births, except those shown with persistent Q1

^e^Multinomial logistic regression, adjusted for substance use within 1 to 365 days before the first birth hospitalization, number of pre-birth comorbidities within 1 to 365 days before the first birth hospitalization (≥ 3 vs. 0–2), age at the first birth hospitalization (15–19, 20–24, 30–34, ≥ 35, vs. 25–29 years), parity (≥ 2 vs. 0–1), residence at the first birth hospitalization (rural vs. urban), immigrant status (non-refugee immigrant vs. non-immigrant), and any newborn congenital or chromosomal anomaly during the first birth hospitalization

^f^An adjusted odds ratio < 1 indicates a lower tendency for downward (or upward) income mobility, and an odds ratio > 1 reflects a higher tendency for such movement

Among mothers initially residing in income Q1, the aOR for remaining in income Q1 was 1.05 (95% CI 1.01–1.08) following a livebirth affected by severe neonatal morbidity, 1.29 (95% CI 1.12–1.48) after a neonatal death, and 1.35 (95% CI 1.24–1.46) after a stillbirth – each compared to mothers with an unaffected livebirth (Table 2).

## Discussion

Mothers with a newborn affected by severe morbidity were more likely to have a decline in neighbourhood income Q, or to persist in the lowest-income area. Those experiencing a stillbirth or neonatal death were more likely to remain in the same neighbourhood income quintile at a subsequent birth (i.e., having no income mobility across births).

Study limitations include a lack of information on a mother’s individual-level income, profession, race, or details about the non-birthing parent, and other unmeasured confounders included in Fig. 1. Individual income changes may not correspond with neighbourhood income mobility. While the current study accounted for livebirth bias at the first birth, survivorship selection bias was likely introduced by the study design, necessitating having a second birth for cohort entry [13]. Maternal peripartum morbidity was not included as a confounder since it is likely a causal mediator of the relation between the study exposure of an adverse perinatal event at the first birth and the ensuing outcome of a mother’s change in residential neighbourhood income quintile [6, 14]. 

We propose that the nature of a pregnancy outcome can be a ‘socioeconomic stress test’ for the parent(s). This stress test may be greater if the newborn experiences severe morbidity: it is plausible that the ongoing requirements of parental care for a newborn affected by morbidity can have both physical, mental, and economic ramifications for one or both parents [15, 16]. In contrast, if the fetus or newborn dies, the economic burden on the family may not persist, despite the emotional burden of that loss.

The exception appears to be among those residing in low-income areas, in which a fetal or neonatal morbidity/loss may profoundly affect her socioeconomic trajectory.

## Conclusions

Future studies should elucidate the causal pathways between having a pregnancy ending in either a stillbirth, neonatal death, or severe morbidity experience and the various patterns of subsequent neighbourhood income movement. Further research should also investigate whether, following an adverse perinatal event, supports such as financial supplements can help a mother at risk of income decline, especially among those already residing in the lowest income areas preceding an adverse perinatal event.

## Supplementary Information

Below is the link to the electronic supplementary material.

Supplementary Material 1

## Data Availability

The dataset from this study is held securely in coded form at ICES. While legal data sharing agreements between ICES and data providers (e.g., healthcare organizations and government) prohibit ICES from making the dataset publicly available, access may be granted to those who meet pre-specified criteria for confidential access, available at www.ices.on.ca/DAS (email: das@ices.on.ca). The full dataset creation plan and underlying analytic code are available from the authors upon request, understanding that the computer programs may rely upon coding templates or macros that are unique to ICES and are, therefore, either inaccessible or may require modification.

## References

[1] Cahn J, Sundaram A, Balachandar R, et al. The association of childbirth with medical debt in the USA, 2019–2020. J Gen Intern Med. 2023;38(10):2340–6. 37199904 10.1007/s11606-023-08214-3 PMC10192781

[2] Zhang X. The post-childbirth employment of Canadian mothers and the earnings trajectories of their continuously employed counterparts, 1983 to 2004. Statistic Canada; 2008.

[3] Taylor K, Compton S, Kolenic GE, et al. Financial hardship among pregnant and postpartum women in the United States, 2013 to 2018. JAMA Netw Open. 2021;4(10):e2132103. 34714338 10.1001/jamanetworkopen.2021.32103 PMC8556621

[4] Machado W, Jaspers E. Money. Birth, gender: explaining unequal earnings trajectories following parenthood. Sociol Sci. 2023;10:429–53.

[5] Jairam JA, Vigod S, Siddiqi A, Guan J, Boblitz A, Wang X, O’Campo P, Ray JG. Morbidity and mortality of newborns born to immigrant and nonimmigrant females residing in low-income neighbourhoods. CMAJ. 2023;195(15):E537–47. 10.1503/cmaj.221711 PMC10110337 37068807

[6] Aoyama K, Park AL, Davidson AJF, Ray JG. Severe maternal morbidity and infant mortality in Canada. Pediatrics 2020;146(3). 10.1542/peds.2019-3870 32817396

[7] Murphy-Kaulbeck L, Belzile N, Tsundu D, Cook JL. Preventing stillbirth in Canada: A need for a coordinated National action plan. J Obstet Gynecol Canada: JOGC = J D’obstetrique Et Gynecologie Du Can: JOGC. 2025;47(Suppl 1):102945. 10.1016/j.jogc.2025.102945 40482708

[8] Flenady V, Boyle F, Koopmans L, Wilson T, Stones W, Cacciatore J. Meeting the needs of parents after a stillbirth or neonatal death. BJOG. 2014;121(Suppl 4):137–40. 25236648 10.1111/1471-0528.13009

[9] Cimpian DM, Strete GE, Cimpian CI, et al. The Impact of preterm birth on parents’ mental health and the role of family-centred interventions: a narrative review. Child (Basel). 2025;12(10). 10.3390/children12101311 PMC12564458 41153493

[10] Jairam JA, Vigod SN, Siddiqi A, et al. Neighborhood income mobility and risk of neonatal and maternal morbidity. JAMA Netw Open. 2023;6(5):e2315301. 37219900 10.1001/jamanetworkopen.2023.15301 PMC10208146

[11] Heazell AEP, Siassakos D, Blencowe H, et al. Stillbirths: economic and psychosocial consequences. Lancet. 2016;387(10018):604–16. 26794073 10.1016/S0140-6736(15)00836-3

[12] Nelson CR, Ray JG, Auger N, et al. Neonatal adverse outcomes among hospital livebirths in Canada: a national retrospective study. Neonatology 2024. 10.1159/000540559 PMC11809516 39173602

[13] Heinke D, Rich-Edwards JW, Williams PL, et al. Quantification of selection bias in studies of risk factors for birth defects among livebirths. Paediatr Perinat Epidemiol. 2020;34(6):655–64. 32249969 10.1111/ppe.12650 PMC7541428

[14] Ray JG, Berger H, Aoyama K, Cook JL, Aflaki K, Park AL. Does infant birthweight percentile identify mothers at risk of severe morbidity? A Canadian population-based cohort study. Matern Health Neonatol Perinatol. 2025;11(1):19. 40604901 10.1186/s40748-025-00217-8 PMC12225049

[15] Cohen E, Horvath-Puho E, Ray JG, et al. Association between the birth of an infant with major congenital anomalies and subsequent risk of mortality in their mothers. JAMA. 2016;316(23):2515–24. 27997654 10.1001/jama.2016.18425

[16] Rotberg B, Horvath-Puho E, Vigod S, Ray JG, Sorensen HT, Cohen E. Increased maternal new-onset psychiatric disorders after delivering a child with a major anomaly: a cohort study. Acta Psychiatr Scand. 2020;142(4):264–74. 32406524 10.1111/acps.13181

