# Supplementary material for: Change in maternal income status following stillbirth, neonatal death and severe neonatal morbidity
Source: Matern Health Neonatol Perinatol. 2026 Jan 21;12:3. doi: 10.1186/s40748-026-00249-8 (PMC12822015; doi:10.1186/s40748-026-00249-8)
Supplement: Supplementary file 1 — Supplementary Material 1 [file 40748_2026_249_MOESM1_ESM.docx]

**Additional File 1: Supplemental Information**

**Table S1.** List of ICES Databases Used in the Current Study

**Table S2.** Cohort Entry and Exclusion Criteria, Methods, and Coding to Identify Study Outcomes

**Supplemental Methods
Supplemental References**

This supplemental material has been provided by the authors to give readers additional information about their work.

**Table S1: List of ICES Databases Used in the Current Study**

| **Dataset name** | **Description** |
| --- | --- |
| Aggregated Diagnosis Groups (ADG) | The Johns Hopkins Adjusted Clinical Groups (ACGs)® system assigns an ICD code to one of 32 diagnosis clusters known as Aggregated Diagnosis Groups (ADG). Individual diseases or conditions are placed into a single ADG based on 5 clinical dimensions: duration of the condition; severity of the condition; diagnostic certainty; etiology of the condition; and specialty care involvement. ICD codes within the same ADG are similar in both clinical criteria and expected need for healthcare resources. Individuals may have multiple diagnoses and belong to multiple ADGs (between zero and 32 ADGs). |
| Canadian Institute for Health Information Discharge Abstract Database (CIHI-DAD) | Captures all in-patient hospital admission records including obstetric deliveries and deaths. Diagnostic codes are based on the *International Statistical Classification of Diseases and Related Health Problems, Tenth Revision, Canada* (*ICD-10-CA*), and procedural codes are based on the *Canadian Classification of Health Interventions (CCI)*. |
| Immigration, Refugees and Citizenship Canada Permanent Residents Database (IRCC-PRD) | Captures demographic information on all international migrants who obtained permanent residency in Canada from January 1985 to March 2023. |
| Linked Delivering Mothers and Newborns (MOMBABY) | Derived from CIHI-DAD, provides linked inpatient hospital admission records of mothers and their infants. |
| National Ambulatory Care Reporting System (NACRS) | Collects data on hospital- and community-based ambulatory care, such as day surgery, outpatient and community-based clinics, and emergency departments. |
| Ontario Health Insurance Plan Claims Database (OHIP) | Contains information about inpatient and ambulatory visits, consultations, and procedures provided to Ontario residents eligible for Ontario's publicly funded health insurance system by fee-for-service health care practitioners (e.g., physicians, optometrists, laboratories for diagnostic tests, etc.). |
| Ontario Mental Health Reporting System (OMHRS) | Collects data on patients in adult-designated inpatient mental health beds. This includes beds in General, Provincial Psychiatric, and Specialty Psychiatric facilities. |
| Narcotics Monitoring System (NMS) | Collects data on dispensed prescriptions for narcotics, controlled substances, and other monitored drugs, irrespective of whether the prescription is paid for under a publicly funded drug program, through private insurance, or by cash. |
| Postal Code Conversion File Plus (PCCF+) | A digital file that links the Canada Post Corporation (CPC) six-character postal code and Statistics Canada’s standard geographic areas (e.g., dissemination area). Area-level income quintiles range from Q1 (lowest) to Q5 (highest) income neighbourhoods. |
| Registered Persons Database (RPDB) | Includes vital status and sociodemographic information about all individuals who have ever received an Ontario Health Insurance Plan (OHIP) number (e.g., date of birth, sex, and postal code). |
| Same Day Surgery (SDS) | Contains demographic, diagnostic, procedural, and treatment information about all day surgical procedures. |
| Statistic Canada Census | Information from the Canadian Census, statistical information about the population including population counts and various levels of geography (e.g., census metropolitan areas, communities, census tracts, etc.) |

**Table S2: Cohort Entry and Exclusion Criteria, Methods, and Coding to Identify Study Outcomes**

| **Assessment** | **Timing** | **Disease, procedure, or condition** | **ICD-10-CA or CCI codes in CIHI-DAD, SDS, and NACRS** | **Other sources** |
| --- | --- | --- | --- | --- |
| ***Inclusion criteria*** | April 1, 2003 to March 31, 2023, at the time of the mother's index delivery hospitalization date, for the first and second births in the study cohort | - All hospital singleton livebirths and stillbirths at 20^0/7^ to 42^0/7^ weeks’ gestation among women residing in Ontario at the time of their first birth during the study period, and who had a second consecutive birth in Ontario. - Among mothers with more than two births in the study period, an earlier livebirth or stillbirth was randomly selected to serve as the *first birth* in the cohort. A consecutive livebirth or stillbirth served as the *second birth*. - Births were limited to women aged 15 to 50 years old with a valid OHIP/IKN number from MOMBABY for the first and second index delivery hospitalizations, and who had a valid postal code recorded at each birth. | MOMBABY | RPDB |
| ***Exclusion criteria applied to all birth records*** | At the mother’s index delivery hospitalization for the first & second births | - Records with warning for mother's IKN or KEY | MOMBABY/RPBD: warning for IKN/KEY (WARN not =”N”(No Warning)). Include N=no warning | -- |
|  | Same as above | - Multiple births | MOMBABY (M_MULTIBIRTH=’T’ or B_MULTIBIRTH=’T’) | -- |
|  | At the mother’s index delivery hospitalization for the first & second births | - Infant gestational age at birth is <20 or ≥43 weeks’ gestation or gestational age missing | MOMBABY: a. B_GESTWKS_DEL not in MOMBABY b. gestational age <20 or ≥43 weeks’ gestation | -- |
|  | At the mother’s index delivery hospitalization for the first & second births | - Women aged < 15 or > 50 years, or age missing | MOMBABY/RPDB:  M_IKN age < 15 or > 50 or missing | -- |
|  | Same as above | - Women who had an invalid OHIP number or hospital number | MOMBABY/RPDB:  Invalid M_IKN (maternal IKN)  a. VALIKN ne ‘V’  b. M_IKN not in RPDB (according to no sex and no bdate)  c. M_IKN with sex =’M’ in RPDB | -- |
|  | Same as above | - Women who were a non-Ontario resident | -- | RPDB: M_IKN non-Ontario resident (substr (prcddablk, 1,2 ne ‘35’) |
|  | Same as above | - Women missing their residential postal code/neighbourhood income quintile |  | RPDB, PCCF+, Statistics Canada census data |
|  | Same as above | - Rural/urban residence missing | -- | RPDB, PCCF+ |
|  | At the time of arrival in Canada | - Immigrants with a landing date before their birth date | RPDB | IRCC-PRD |
|  | Same | - Refugees and other immigrants | -- | IRCC-PRD |
|  | At the time of arrival in Canada | - Women concurrently recorded as a non-refugee immigrant & Canadian-born | -- | IRCC-PRD |
| ***Exclusion criteria applied to the first selected livebirth or stillbirth during the study period*** | At the mother’s index delivery hospitalization for the first birth | Women without a second consecutive birth during the study period | MOMBABY | *--* |
| ***Exclusion criteria applied to the second selected livebirth or stillbirth during the study period*** | At the time of arrival to Canada & the mother’s delivery hospitalization date for the second birth | Immigrants with a landing date after their admission date for the second birth | RPDB | IRCC-PRD |
|  | At the mother’s index delivery hospitalization for the first & second births | < 161 days between these dates | MOMBABY: B_BDATE | -- |
| ***Study exposure – Stillbirth, neonatal death and severe neonatal morbidity*** | At the infant’s index birth hospitalization for the first birth | Adverse pregnancy outcomes (4-level) categorical variable:   1. livebirth unaffected by severe neonatal morbidity or neonatal death (referent); 2. livebirth with severe neonatal morbidity and no neonatal death, arising in the index birth admission or within 27 days thereafter; 3. livebirth with neonatal death, arising in the index birth admission or within 27 days thereafter; 4. stillbirth: fetal death arising *in utero*, or a newborn with no signs of life at birth, at ≥ 20 weeks’ gestation -- among livebirths and stillbirths.   *Note for the exposure groups ii) and iii) if the birth hospitalization length of stay was: ≤ 27 days, then these events were further assessed within any subsequent; rehospitalization, up to 27 days after birth.  > 27 days, then these events were assessed any time during that hospitalization, even if it surpassed 27 days. | Severe neonatal morbidity – among livebirths:  Birth Trauma P10.0 to P10.3, P13.0, P13.2, P13.3, P14.0, P14.1 P14.2, P14.3, P14.8, P14.9, P13.4  Necrotising enterocolitis P77  Seizure P90, R56  Intraventricular haemorrhage P52.1, P52.2  Cerebral infarction I63  Periventricular leukomalacia P91.2 | Severe neonatal morbidity based on validated the Canadian NAOI^a^ |
|  |  |  | Hypoxic ischaemic encephalopathy P91.5, P91.6, P91.8  Infection P36 G00-03, G05, A40, A41.5, A41.8, A41.9, B95.1, B96.2  Respiratory distress syndrome P22.0  Chronic respiratory disease originating in the perinatal period  P27  Pneumonia  P23, J12-J18  Other respiratory  P28.0, P28.5  Perinatal intestinal perforation  P78.0  Retinopathy of prematurity  H35.1  Ventilatory support 1GZ31CAEP, 1GZ31CAND, 1GZ31CAPK, 1GZ31CBND, 1GZ31CRND, 1GZ31GPND, 1GZ31JAMD, 1GZ31JANC, 1GZ31JAPK  Pneumothorax requiring intercostal catheter 1GV52DA, 1GV52DATS, 1GV52HA, 1GV52HAHE, 1GV52HATK, 1GV52LA, 1GV52LATS, 1GV52LAXXE, 1GV54JATS, 1GV55JATS |  |
|  |  |  | Any body cavity surgical procedure 1AA52, 1AA87, 1AC87, 1AE87, 1AF87, 1AG87, 1AJ87, 1AK87, 1AN52, 1AN59, 1AN87, 1AP59, 1AP72, 1AP87, 1AW59, 1AW72, 1AW87, 1AX87, 1BA72, 1BA80, 1BA87, 1BB72, 1BB80, 1BB87, 1BD72, 1BD80, 1BD87, 1BF80, 1BG72, 1BG80, 1BG87, 1BK59, 1BM72, 1BM80, 1BM87, 1BN72, 1BN80, 1BN87, 1BP72, 1BP80, 1BP87, 1BQ72, 1BQ80, 1BQ87, 1BS72, 1BS80, 1BS87, 1BT72, 1BT80, 1BT87, 1GA87, 1GA89, 1GB87, 1GB89, 1GD89, 1GE80, 1GE87, 1GE89, 1GE91, 1GH84, 1GJ86, 1GJ87, 1GK87, 1GK89, 1GM80, 1GM86, 1GM87, 1GN92, 1GR87, 1GR89, 1GR91, 1GT78, 1GT87, 1GT89, 1GT91, 1GV87, 1GV89, 1GW87, 1GX80, 1GX86, 1GX87, 1GY70, 1GY72, 1GY86, 1HJ76, 1HJ82, 1HN87, 1HP76, 1HP78, 1HP80, 1HP82, 1HP83, 1HP87, 1HR80, 1HR84, 1HR87, 1HS80 (excl. 1HS80G), 1HS90, 1HT80 (excl. 1HT80G), 1HT89, 1HT90, 1HU80 (excl. 1HU80G), 1HU90, 1HV80 (excl. 1HV80G), 1HV90, 1HW78, 1HW79, 1HX80, 1HX87, 1HX80, 1HZ87, 1IA76,  1IA80, 1IA87, 1IB76, 1IB79, 1IB80, 1IB82, 1IB87, 1IC76, 1IC80, 1IC82,  1IC87, 1ID76, 1ID80, 1ID82, 1ID86, 1ID87, 1IF83, 1IJ76, 1IJ80, 1IM76, |  |
|  |  |  | 1IM80, 1IM82, 1IM83, 1IM87, 1IN83, 1IN84, 1IN87, 1JE57 (excl. 1JE57G), 1JE76, 1JE80, 1JE87, 1JJ76, 1JJ80, 1JK76, 1JK80, 1JK87, 1JW51 (excL. 1JW51G), 1JW57, 1JW76, 1LA84, 1LC84, 1LD84, 1NA72, 1NA74, 1NA76, 1NA77, 1NA80, 1NA84, 1NA86, 1NA87, 1NA88, 1NA89, 1NA90, 1NA91, 1NA92, 1NE80, 1NF76, 1NF78, 1NF80, 1NF82, 1NF84, 1NF86, 1NF87 (excl. 1NF87B), 1NF89, 1NF90, 1NF91, 1NF92, 1NK76, 1NK77, 1NK80, 1NK82, 1NK84, 1NK87 (excl. 1NK87B), 1NM74, 1NM76, 1NM77, 1NM80, 1NM82, 1NM87 (excl. 1NM87B), 1NM89, 1NM91, 1NP72, 1NP73, 1NP86, 1NQ74 (excl. 1NQ74B), 1NQ80, 1NQ84, 1NQ86, 1NQ87 (excl. 1NQ87B), 1NQ89, 1NQ90, 1NT80, 1NT84, 1NT86, 1NT87, 1NV89, 1OA87, 1OB87, 1OB89, 1OD76, 1OD89, 1OE76, 1OE80, 1OE89, 1OJ76 (excl. 1OJ76B), 1OJ87, 1OJ89, 1OK87, 1OK89, 1OK91, 1OT72, 1OT87, 1OT91, 1PB87, 1PB89, 1PC80, 1PC87 (excl. 1PC87D), 1PC89, 1PC91, 1PE57 (excl. 1PE57BD), 1PE80 (excl. 1PE80D), 1PE82, 1PE87 (excl. 1PE87D), 1PE89 (excl. 1PE89D), 1PG76, 1PG77, 1PG80 (excl. 1PG80D), 1PG86, 1PG89, 1PL74 (excl. 1PL74CD), 1PL80, 1PM79, 1PM86, 1PM87 (excl. 1PM87B), |  |
|  |  |  | 1PM89, 1PM90, 1PM91, 1PM92, 1QE53, 1QE80, 1QE82, 1QE84, 1QE87, 1QE89, 1QG89, 1QM74, 1QM80, 1QM87, 1QM89, 1QM91, 1QN82, 1QT87, 1QT91, 1RB74, 1RB80, 1RB83, 1RB87, 1RB89,1RD89, 1RF51, 1RF72, 1RF74, 1RF80, 1RF87, 1RF89, 1RM87 (excl. 1RM87B), 1RM89, 1RM91, 1RN87, 1RN89, 1RS74, 1RS80, 1RS86, 1RS87, 1RS89, 1RW87, 1RW88, 1RW91, 1RW92, 1SA74, 1SA75, 1SA80, 1SA89, 1SC74, 1SC75, 1SC80, 1SC87, 1SC89, 1SE53, 1SE89 (excl. 1SE89D), 1SF80, 1SF87, 1SF89, 1SG80, 1SG87, 1SH87, 1SM74, 1SM80, 1SM87, 1SN87, 1SN93, 1SQ53, 1SQ74, 1SQ80, 1SQ87, 1SQ91, 1SQ93, 1SW74, 1SY80, 1SY84, 1SY87, 1SZ87, 1VA53, 1VA74, 1VA75, 1VA80, 1VA87, 1VA93, 1VC74, 1VC80, 1VC87, 1VC91, 1VC93, 1VE80, 1VG53, 1VG55, 1VG72, 1VG73, 1VG74, 1VG75, 1VG80, 1VG87, 1VG93, 1VK80, 1VK87, 1VK89, 1VL80, 1VL87, 1VM80, 1VM87, 1VN80, 1VN87, 1VP74, 1VP80, 1VP87, 1VP89, 1VQ74, 1VQ79, 1VQ80, 1VQ82, 1VQ87, 1VQ91, 1VQ93, 1VS72, 1VS80, 1VX87  Resuscitation by intubation and/or chest compressions  1.GZ.30^^ 1.HZ.30^^ |  |
|  |  |  | Central venous or arterial catheter insertion  1KV53HACH, 1KV53HAFT, 1KV53LAFT, 2IM28GP, 2LZ28GQPL, 2LZ28GRPL, 2LZ28JAPL, 1KX53HACH, 1KX53HAFT*, 1KX53LAFT*, 2LZ28GQPL, 2LZ28GRP, 1IS53^^ * Up until 2015 umbilical venous catheterization (UVC) was classified in these codes. After 2015, they were found in 1IS53^^.  Administration of any intravenous fluid 1.LZ.35^^  Transfusion of red blood cells or a blood product 1LZ19HHU1A, 1LZ19HHU1J, 1LZ19HHU2A, 1LZ19HHU2J, 1LZ19HHU3J, 1LZ19HHU4J, 1LZ19HHU5J, 1LZ19HHU6A, 1LZ19HHU6J, 1LZ19HHU9A, 1LZ19HHU9J, 1LZ19HMU1, 1LZ19HMU2, 1LZ19HMU9, 1LZ35HAC5  Note: CIHI has a “BTANY” variable which also summarizes all the above |  |
|  | Same | All-cause neonatal mortality – among livebirths | -- | RPDB |
| ***Study outcome*** | At the mother’s index delivery hospitalization for the first and second consecutive births | A change in maternal residential neighbourhood income quintile (Q) between the two consecutive births – **4 exclusive income mobility patterns**:  i) **D*ownward income mobility –*** moving from a higher income Q area (i.e., Q2, 3, 4, or 5) to any lower income Q area (i.e., Q1, 2, 3, or 4) between two consecutive births ii) **U*pward income mobility –*** moving from a lower (i.e., Q1, 2, 3, or 4) to a higher income Q area (i.e., Q2, 3, 4, or 5) between two consecutive births  iii) ***Persistently residing in income Q1* *–*** women who remained in Q1 at each birth iv) ***No change in neighbourhood income quintile –*** (referent): remaining in the same income Q area (i.e., Q2, 3, 4, or 5) between two consecutive births | -- | RPDB, PCCF+, Statistic Canada census data  *A mother's postal code at each birth and census area-level income data were used to derive neighbourhood income Q. |
| ***Descriptive variables and covariates*** | 1 to 365 days prior  to the mother’s delivery hospitalization for the first birth | Number of pre-birth comorbidities: Total number of Aggregated Diagnosis Groups (ADGs), excluding any pregnancy defined ADG. Operationalized as: 0-2 or ≥ 3 comorbidities. | ADGs are obtained from diagnosis codes in DAD, SDS, and NACRS using The Johns Hopkins ACG® System Version 10 software | -- |
|  | Same | Substance use: any documented use of opioids, cocaine, stimulants, or alcohol. | Any opioid use: based on prenatal prescription opioid data, outpatient visits for opioid agonist therapy (OAT), and/or opioid-related hospital records   - DAD/NACRS: Any opioid exposure - F11, T40.0-T40.4, T40.6, Y45.0   Any cocaine use: DAD or NACRS  F14, T40.5, R78.2  Any stimulant use: DAD or NACRS  F15  Any alcohol use: DAD or NACRS Z72.1 | Any opioid use: OHIP: Codes K682, K683, K684   - NMS: Any prescriptions for opioid agents - OHMRS DSM 4/5: Diagnosis codes 304.00, 305.50 |
|  | At the mother’s index delivery hospitalization for the first birth | Neighbourhood income quintile (Q1, 2, 3, 4, or 5) at the first birth | -- | RPDB, PCCF+, Statistic Canada census data |
|  | At the mother’s index delivery hospitalization for the first birth | Maternal age (years) | MOMBABY: M_AGE1 | -- |
|  | Same | Livebirth parity: number of previous livebirths (Previous term deliveries + Previous preterm deliveries), operationalized as: 2 or more livebirths vs. 0-1 livebirths. | CIHI-DAD/MOMBABY | -- |
|  | Same | Rural vs. urban residence | -- | RPDB, PCCF+ |
|  | At the time of arrival in Canada | Immigration status: non-refugee immigrants vs. non-immigrants. (An immigrant woman must have been born outside of Canada and then migrated to, and obtained permanent residency, in Ontario). | -- | IRCC-PRD |
|  | Same | Infant’s biological sex assigned at birth | MOMBABY: b_sex | -- |
|  | Same | Gestational age at birth, in weeks | MOMBABY | -- |
|  | Same | Preterm birth liveborn or stillborn < 37 weeks’ gestation | MOMBABY | -- |
|  | Between 0 days (at the newborn’s birth hospitalization [first birth]) & up to the hospitalization discharge date (first birth) | Any congenital or chromosomal anomaly – among livebirths | ICD-10 Q00-Q99 | -- |
|  | The newborn’s birth admission dates, for first and second births | Interpregnancy birth interval: Time elapsed between the infant’s birth admission, for first and the second births (in months). Calculated by subtracting the infant’s birth admission date for the first birth from the infant’s birth admission date for the second birth. | MOMBABY: B_BDATE | -- |

^a^Nelson CR, Ray JG, Auger N, et al. Neonatal Adverse Outcomes Among Hospital Livebirths in Canada: A National Retrospective Study. *Neonatology.* 2024.

Abbreviations: ACG: Adjusted Clinical Group; ADG: Aggregated Diagnosis Group; CIHI: Canadian Institute for Health Information; DAD: Discharge Abstract Database; CCI: Canadian Classification of Health Interventions; ICD-10-CA: International Classification of Diseases, 10th Revision, Canada; IRCC-PRD: Immigration, Refugees, and Citizenship Canada Permanent Resident Database; NACRS: National Ambulatory Care Reporting System; NMS: Narcotics Monitoring System; OHIP: Ontario Health Insurance Plan; OMHRS: Ontario Mental Health Reporting System; ORG-D: Office of Registrar General – Deaths; PCCF+: Postal code conversion file plus (Statistics Canada); RPDB: Registered Persons Database; SDS: Same Day Surgery Database.

**Supplemental Methods**

*Study Design, Settings, and Participants*

This retrospective population-based cohort study was completed using linked administrative health datasets in Ontario, Canada (Table S1), where universal healthcare exists. Included were all females with two consecutive singleton hospital-based livebirths or stillbirths at 20 to 42 weeks’ gestation, between April 1, 2003 and March 31, 2023. For those who had more than two births during the study period, an earlier livebirth or stillbirth was randomly selected to serve as the *first birth* in the cohort. The next livebirth or stillbirth was assigned as the *second birth*. The cohort was limited to females aged 15 to 50 years, who had a valid Ontario Health Insurance Plan (OHIP) number and residential postal code at both births (Table S2).

The use of deidentified data in this project was authorized under section 45 of Ontario’s Personal Health Information Protection Act and exempt from a research ethics board review and informed consent). The [STROBE](http://www.equator-network.org/reporting-guidelines/strobe/) reporting guideline was followed.

*Data Sources*

Multiple health administrative databases were linked via unique encoded identifiers and stored and analyzed at ICES. If a mother did not have a valid unique encoded identifier, database linkages could not be completed, and she was excluded from the study cohort. ICES is an independent, non-profit research institute, whose legal status under Ontario’s health information privacy law allows it to collect and analyze health care and demographic data, without consent, for health system evaluation and improvement. All datasets used for this study are valid and reliable sources for perinatal research (Table S1).^1-3^ All maternal-newborn pairs were identified in the ICES derived database named “MOMBABY”, which captures nearly all births (98%) in Ontario. Datasets are shown in Table S1.

*Study Exposure*

The study exposure ─ an adverse perinatal outcome -- comprised four mutually exclusive groups, ascertained at the **first** of the two consecutive births in the study period: i) livebirth unaffected by severe neonatal morbidity or neonatal death (referent); ii) livebirth with severe neonatal morbidity and no neonatal death; iii) livebirth with neonatal death; and iv) stillbirth ─ a fetal death arising *in utero*, or a newborn with no signs of life at birth, at ≥ 20 weeks’ gestation. Severe neonatal morbidity and neonatal death were assessed from the index birth hospitalization up to 27 days thereafter.

Severe neonatal morbidity was assessed using a Canadian-validated version of the Neonatal Adverse Outcomes Indicator (NAOI)^4^ that includes a total of 22 components ─ 15 neonatal complications (e.g., birth trauma, seizures) recorded using *International Statistical Classification of Diseases and Related Health Problems, Tenth Revision, Canada (ICD-10-CA)* diagnostic codes,^5^ and 7 interventions (e.g., resuscitation by intubation and/or chest compressions) recorded using the *Canadian Classification of Health Interventions (CCI)*.^6^ The Canadian NAOI identifies newborns who experience a severe complication during the birth hospitalization or the neonatal period, and it is associated with a higher risk of neonatal death, admission to a neonatal intensive care unit, and prolonged hospital stay.^4^

In the current study, severe neonatal morbidity was defined as present if a newborn had 1 or more NAOI components arising in their index birth hospitalization, and absent if no NAOI components were recorded during their index birth hospitalization. If a newborn’s index birth hospitalization length of stay was ≤ 27 days, they were further assessed within any subsequent rehospitalization, up to 27 days after birth. If a newborn’s index birth hospitalization duration was > 27 days, they were assessed any time during that hospitalization, even if it surpassed 27 days. The same approach was used to capture neonatal death at the index birth hospitalization and within 27 days thereafter.

All study variables are otherwise detailed in the online Supplement Table S2.

*Study Outcome*

The study outcome was a mother’s change in residential neighbourhood income quintile (Q) between two consecutive births, representing four exclusive mobility patterns: i) downward income mobility - moving from a higher income Q area (i.e., Q2, 3, 4, or 5) to any lower income Q area (i.e., Q1, 2, 3, or 4) between births or ii) upward income mobility ***–*** moving from a lower income Q area (i.e., Q1, 2, 3, or 4) to a higher income Q area (i.e., Q2, 3, 4, or 5) between births, or iii) persistently residing in income Q1 at each birth -- each relative to iv) no change in neighbourhood income Q between births (referent), excluding those persistently residing in income Q1. The latter two exposure groups were categorized separately as they likely comprise mothers with different economic circumstances, especially that persistent residence in a lowest income neighbourhood would be is indicative of long-term area-level economic instability.

Neighbourhood income Q is an area-level measure of socioeconomic position that can change over an individual’s course.^7^ In this study, it refers to the average income per single-person equivalent in a dissemination area, adjusted for household size, and it is obtained from census area-level income data.^8,9^ Dissemination areas are the smallest unit of census geography, including roughly 400 to 700 people,^10^ and following natural urban boundaries and census divisions.^11^

The six-character residential postal code recorded at the mother’s first and second delivery hospitalizations was used to determine her neighbourhood income Q at each birth. This method is explained in a prior study.^12^ A postal code boundary parallels a dissemination area, herein, referred to as a “neighbourhood”.

*Statistical Analysis*

Descriptive statistics (i.e., mean and median values and proportions) were contrasted by the adverse pregnancy outcome exposure states at the first birth. Multinomial logistic regression generated odds ratios (aORs) and 95% confidence intervals (CI) for the primary outcome of a mother’s change in neighbourhood income Q between two consecutive births in relation to the study exposure of an adverse pregnancy outcome at the first birth.

Statistical models were adjusted for the following maternal and newborn characteristics: substance use (opioid, cocaine, stimulant, or alcohol use) within 1 to 365 days before the first birth hospitalization; number of prebirth comorbidities within 1 to 365 days before the first birth hospitalization, assessed using the Johns Hopkins Adjusted Clinical Group (ACG)® System (version 10 software), excluding pregnancy-related, and categorized into Aggregated Diagnosis Groups (≥ 3 vs. 0-2);^13^ maternal age (15-19, 20-24, 30-34, ≥ 35 vs. 25-29 years) at the first birth hospitalization; livebirth parity (≥ 2 vs. 0-1) at the first birth hospitalization; residence (rural vs. urban) at the first birth hospitalization; immigrant status (non-refugee immigrant vs. non-immigrant); and any newborn congenital or chromosomal anomaly identified within the first birth hospitalization. A non-refugee immigrant woman was born outside of Canada and then migrated to, and obtained permanent residency, in Ontario. Immigration records are available for a non-refugee immigrant within the Immigration, Refugees and Citizenship Canada Permanent Resident.
 Data were analyzed from December 2024 to January 2025, using SAS version 9.4 (SAS Institute). The magnitude of OR and precision of corresponding 95% CI indicated significant differences between exposure groups.

**Supplemental References**

1. Wen SW, Liu S, Marcoux S, Fowler D. Uses and limitations of routine hospital admission/separation records for perinatal surveillance. *Chronic Dis Can.* 1997;18(3):113-119.

2. Joseph KS, Fahey J, Canadian Perinatal Surveillance S. Validation of perinatal data in the Discharge Abstract Database of the Canadian Institute for Health Information. *Chronic Dis Can.* 2009;29(3):96-100.

3. Juurlink D PC, Croxford R, Chong A, Austin P, Tu J, et al. . *Canadian Institute for Health Information Discharge Abstract Database: A Validation Study.* Toronto: ICES;2006.

4. Nelson CR, Ray JG, Auger N, et al. Neonatal Adverse Outcomes Among Hospital Livebirths in Canada: A National Retrospective Study. *Neonatology.* 2024.

5. Canadian Institute for Health Information. *Final report. The Canadian enhancement of ICD-10 (International Statistical Classification of Diseases and Related Health Problems, tenth revision).* Ottawa: Canadian Institute for Health Information;2001.

6. Canadian Institute for Health Information. *International Statistical Classification of Diseases and Related Health Problems, tenth revision. CCI/ICD-10.* Ottawa Canadian Institute for Health Information;2010.

7. Shavers VL. Measurement of socioeconomic status in health disparities research. *Journal of the National Medical Association.* 2007;99(9):1013-1023.

8. Canadian Institute for Health Information. *Trends in Income-Related Health Inequalities in Canada: Methodology Notes.* Ottawa, ON: CIHI;2015.

9. Statistics Canada. Postal Code^OM^ Conversion File Plus (PCCF+) Version 8A, Reference Guide. December 2022 Postal codes. <https://guides.library.queensu.ca/ld.php?content_id=34898339>. Accessed June 15, 2024.

10. Statistics Canada. Dissemination Area. In. Ottawa, ON: Statistics Canada; 2021.

11. Buajitti E, Rosella LC. Neighbourhood socioeconomic improvement, residential mobility, and premature death: a population-based cohort study and inverse probability of treatment weighting analysis. *International journal of epidemiology.* 2022.

12. Jairam JA, Vigod SN, Siddiqi A, et al. Neighborhood Income Mobility and Risk of Neonatal and Maternal Morbidity. *JAMA Netw Open.* 2023;6(5):e2315301.

13. John Hopkins. ACG System version 10.0 Technical Reference Guide. <https://www.hopkinsacg.org/document/acg-system-version-10-0-technical-reference-guide/>. Accessed May 22, 2024.
